# Modeling and Control of Layer Height in Laser Wire Additive Manufacturing

**DOI:** 10.3390/ma15134479

**Published:** 2022-06-25

**Authors:** Natago Guilé Mbodj, Mohammad Abuabiah, Peter Plapper, Maxime El Kandaoui, Slah Yaacoubi

**Affiliations:** 1 Department of Engineering, University of Luxembourg, 6, Rue -Kalergi, L-1359 Luxembourg, Luxembourg; natago.mbodj@uni.lu (N.G.M.); peter.plapper@uni.lu (P.P.); 2 Mechanical and Mechatronics Engineering Department, Faculty of Engineering and Information Technology, An-Najah National University, Nablus P.O. Box 7, Palestine; 3 Plateforme DRIEG CND and Assembly, Institut de Soudure, 4 Bd Henri Becquerel, 57970 Yutz, France; m.elkandaoui@isgroupe.com (M.E.K.); s.yaacoubi@isgroupe.com (S.Y.)

**Keywords:** Laser Wire Additive Manufacturing, Model Predictive Controller, physics-based model

## Abstract

Laser Wire Additive Manufacturing (LWAM) is a flexible and fast manufacturing method used to produce variants of high metal geometric complexity. In this work, a physics-based model of the bead geometry including process parameters and material properties was developed for the LWAM process of large-scale products. The developed model aimed to include critical process parameters, material properties and thermal history to describe the relationship between the layer height with different process inputs (i.e., the power, the standoff distance, the temperature, the wire-feed rate, and the travel speed). Then, a Model Predictive Controller (MPC) was designed to keep the layer height trajectory constant taking into consideration the constraints faced in the LWAM technology. Experimental validation results were performed to check the accuracy of the proposed model and the results revealed that the developed model matches the experimental data. Finally, the designed MPC controller was able to track a predefined layer height reference signal by controlling the temperature input of the system.

## 1. Introduction

Metallic Additive Manufacturing (M-AM) process development began in the late 1990s [1,2]. The process consists of melting a wire or powder using an energy source to create a liquid melt pool bead. The beads are then added layer by layer to form the part. Today’s application of the process can be found in the automotive sector, aircraft, medical implants, dental restoration, and even the fashion sector [3,4,5]. M-AM integration in the industrial sector is still in process because of the complexity and the interference of highly sensitive parameters causing disturbances. A fluctuation of one process parameter such as the wire-feeding rate, power, and deposition speed can modify the melt pool shape, thus to the final part quality and integrity [6,7].

According to different sources of energy used for metal deposition, M-AM could be classified into Arc Additive Manufacturing (AAM), Electron Beam Additive Manufacturing (EBAM), and Laser Additive Manufacturing (LAM) [8]. With regard to the additive material form, Laser Additive Manufacturing could be divided into powder-based and wire-based Laser Additive Manufacturing. In this paper, we will focus on the Laser Wire Additive Manufacturing (LWAM) technology.

**Figure 1:**
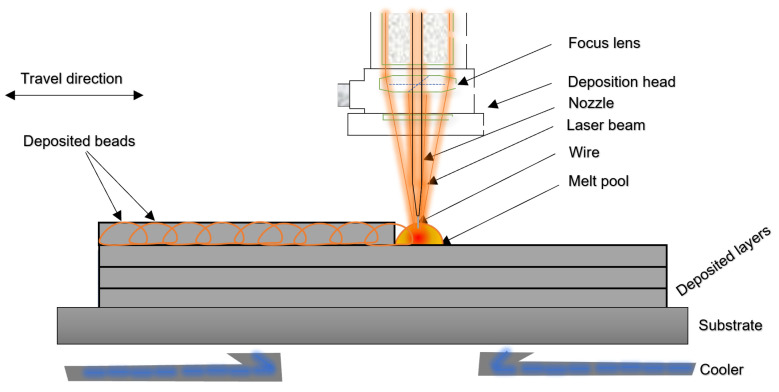
Schematic diagram for bead deposition process in the LWAM.

The process of LWAM consists of melting a wire using an energy source to create a liquid melt pool bead. The beads are then added layer by layer to form the final object, as illustrated in Figure 1.

To obtain a stable deposition process, reliable sensing, modeling and control approaches are needed. In Mbodj et al. [9], a model to predict bead geometry and improve deposition accuracy was proposed. More specifically, a regression algorithm is applied to fit bead geometry with the main deposition process parameters (laser power, wire feed rate and advanced speed) and a neural network-based approach was used to study the influence of the parameters on the bead geometry. Magerramova et al. [10] investigated body parts using computational and experimental methods. The Finite Element Method (FEM) was used in the Laser-Based Manufacturing (LBM) process to simulate gearbox housings and numerically optimize the product weight. The results show that the mass of body parts is reduced by up to 15% with the same strength properties. Finally, Liu et al. [11] used a 3D microscale FEM with a powder arrangement system to simulate multi-layer powder stacking for Selective Laser Sintering (SLS). The model aimed mainly to investigate the thermal evolution of selective laser sintering using metal powders.

Furthermore, Fetni et al. [12], developed an empirical model to study the impact of process parameters on the deposition. The thermal history and the melt pool dimension evolution of a 316L stainless steel reinforced by Tungsten carbides were studied using finite elements. The experimental analysis was correlated with the numerical results using experimental observations from light optical, scanning electron microscopies and thermocouple records. Corbin et al. [13], proposed an empirical model of single bead geometry and the process parameters was developed. Linear regression was applied to fit the collected data using an optical profilometer. The response variables are the bead height, bead width, and angle of repose. The model showed the influence of the process parameter’s interaction on the bead geometry. Kiran et al. [14] developed a thermo-mechanical weld model for 316L stainless steel. Their model estimates the residual stress for large parts. The results obtained are compared with the experimentally measured thermal field to validate the approach. On the other hand, Gockel et al. [15] created a process map of single beads of Ti-6Al-4V using finite element analysis. The process is developed for the microstructure solidification in electron beam wire feed AM processes.

The presented computational methods, for developing a physical model of the bead deposition, are limited and complicated to be employed from the control system design point of view. Therefore, an analytical model is suggested for process optimization as well as for designing a stable and fast controller. The first analytical model for metal deposition was developed in 2001 by Doumanidis et al. [16]. The analytical model includes the material transfer phenomena and thermal transfer of the moving source. The model was derived from the molten pool’s scalar mass, momentum, thermal conduction, and energy balances. Years later, Wang et al. [17] proposed an improved model based on Doumanidis et al.’s work. The developed physics-multivariable model proposed a parameterization of the material transfer rate as a function of the process parameters, thus characterizing the steady-state melt-pool geometry. Yuze et al. [18] proposed an analytical method to predict the clad geometry and the catchment efficiency. The model couples the moving laser beam, the powder stream, the semi-infinite substrate with the heated powder spatial distribution, the attenuated laser power distribution, and the 3D shape of the melt pool. Furthermore, Yuze et al. [19] developed a physics-based model for melt pool dimensions, height, width, and wetting angle. The model was applied on single-track to multi-track and multi-layer deposition. The experimental validation showed good agreement at different levels of specific energy and powder feed rate for single-track simulation and good prediction of the dynamic height for 3D profile structures. However, some discrepancies between the model prediction and the experimental results were noticed. The differences came from the deviation of the powder feeding and the heat convection leading to oxidation in the process.

Regarding the monitoring systems and controller design in the M-AM, Farshidianfar et al. [20] developed an infrared system to monitor surface temperatures of deposition microstructure during Laser Additive Manufacturing (LAM). A PID feedback controller stabilizes the cooling rate by adjusting the travel speed. The experimental results show that the controller can achieve acceptable microstructure in real-time. In the work of Garmendia et al. [21], a structured light scanner is used to obtain the part height. A closed-loop controller is implemented to adjust the deposition height to ensure a good geometrical accuracy of the final part. The results show that the model can give satisfactory results both on power and wire-based laser metal deposition (LMD). Heralic et al. [22] used a 3D scanning system to obtain the surface for each deposited layer. An iterative learning control system compensates for the depositions by varying the wire feed rate across layers. The experimental results showed that the developed model works for the automatic deposition of structures. Gibson et al. [23] presented multiple modes of closed-loop melt pool size control in laser-wire based DED. A real-time closed-loop melt pool size control through laser power modulation and a controller that modulates the print speed and deposition rate on a per-layer basis was developed and demonstrated. In [24], Liu et al. used a near-infrared monochrome (NIRM) camera to get the melt pool size and a first-order transfer function created for an automatic control system. The laser power was used as the input variable in the system and a Model Predictive Controller was created to control the melt pool size. The experimental results showed that the control system had improved the final part quality. Xiong et al. [25] created a single-neuron self-adjusting controller for the wire and arc additive manufacturing (WAAM) process. The controller takes the travel speed as the input to correct the layer width. The experimental results show that the controller contributes to the stability of the layer width. In [26], the clad height is controlled using the scanning speed as the control input. A charge-coupled camera gets the training data profiles and Adaptive Neuro-Fuzzy Inference Systems (ANFIS) is developed to control the system. The experimental results showed satisfactory outcomes in the laser cladding process. Finally, Zeinali et al. [27] presented a real-time acquisition and control of the clad height. The substrate velocity is taken as the input of the system. A camera is used to obtain the clad height and an adaptive sliding mode control with an uncertainty estimator is implemented. The experimental results showed an improvement in the final deposition.

Based on the cited works, physical model implementation and control design for the LWAM process has not been developed in detail due to system complexity and parameter diversity. Therefore, the aim of this work is to derive a physics-based multi-variable model that describes the LWAM process and to design a Model Predictive Controller (MPC) based on the derived model. More specifically, the proposed model describes the relationship between the layer height and the molten pool behaviour, material properties, process parameters, and thermal history. The proposed model provides easiness and computation efficiency for offline simulation when dealing with process parameters and designing a layer height controller for LWAM. Thus, the main contributions of this work are, (i) proposing a dynamical model description of layer height deposition in the LWAM by taking into account different process parameters and material properties; (ii) designing an MPC to control the layer height to achieve good deposition and improving the quality of the final printed part in the LWAM process.

The rest of this paper is structured as follows, Section 2 and Section 3 provide a detailed theoretical background for the numerical model implementation and MPC design for the LWAM process respectively. Section 4 presents the simulation and experimental results and discussion. The paper finishes in Section 5 with conclusions.

## 2. Numerical Model

As a non-linear process, LWAM involves complex interaction to perform a material transfer using wire and laser power. The main process parameters interfering in the process are the laser power, the travel speed, the standoff distance, and the wire feed rate. Some insights based on previous studies (e.g., [16,28,29]) and the authors’ knowledge of the LWAM process are used to develop the desired model. The proposed model provides a physical description of the LWAM process, considering most of the process requirements. This model can also be considered as a pillar for a reliable controller design.

**Figure 2:**
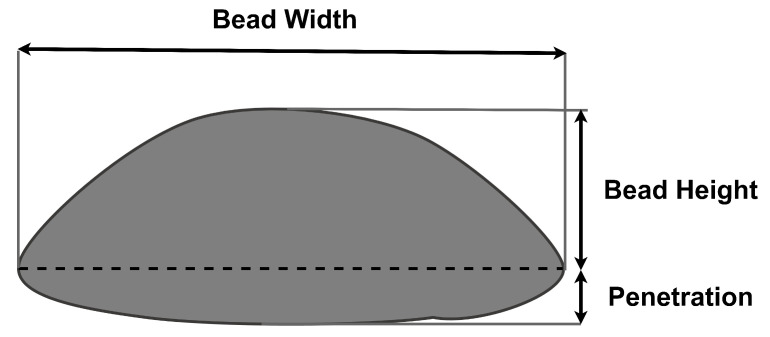
Half-ellipsoidal form of the bead geometry.

Based on Doumanidis et al. [16], the mass change rate of the melt pool is equal to the material transfer rate minus the mass rate of solidification (material loss), and it is given by,

(1) $$\frac{d}{dt}\left(\rho V\right)=\mu f_{r}-\rho Av$$

where ρ is the melt pool density, *V* is the volume of the melt pool, μ is the mass transfer efficiency, $f_{r}$ is the total material transfer rate (wire feed rate), *A* is the cross-section area of the melt pool and *v* is the travel speed. The mass transfer efficiency of the deposition is the ratio of the mass deposited over layers with respect to the consumed wire. Equation (1) was derived based on the approximation of the bead geometry profile to have a half-ellipsoidal form, as shown in Figure 2.

The half of the three-dimensional ellipsoid is characterized by the melt pool width, *w*, the melt pool length, *l*, and the melt pool height, *h*. Therefore, the volume *V* can be expressed with respect to the melt pool geometry variables (*w*, *h* and *l*) such as,

(2) $$V=\frac{\pi }{6}whl$$

and the area *A* is described as,

(3) $$A=\frac{\pi }{4}wh$$

Further, for simplicity, the ratio between the melt-pool width and height will be fixed. Also, the melt-pool width and length are assumed to be equal in the derivation of the equations; in this work, some deposition trials allowed to obtain this ratio for different process parameters, as presented in [9].

It is worth mentioning that, the equation presented by Doumanidis et al. [16] was derived for the gas metal arc welding (GMAW). Thus, a modification is required to reflect and describe the LWAM process properly. Therefore, after substituting the volume, the area, and the the width-length ratio, the mass conservation equation of the melt-pool in the LWAM becomes,

(4) $$\frac{d}{dt}\left(\frac{\pi }{6}\rho whl\right)=\mu f_{r}-\frac{\pi }{4}\rho rh^{2}v$$

The derivative of $\frac{d}{dt}\left(\frac{\pi }{6}\rho whl\right)$ with respect to the melt-pool height variable, assuming that *l* = *w* and the ratio r=w/h (see e.g., [28]), is given by the following equation,

(5) $$\frac{d}{dt}\left(\frac{\pi }{6}\rho wh\left(t\right)l\right)=\frac{d}{dt}\left(\frac{\pi }{6}\rho w^{2}h\left(t\right)\right)=\frac{d}{dt}\left(\frac{\pi }{6}\rho r^{2}h{\left(t\right)}^{3}\right)=\rho \frac{\pi }{2}r^{2}h^{2}\left(t\right)\frac{dh(t)}{dt}$$

Then, the mass balance equation of the melt pool can be rewritten as,

(6) $$\rho \frac{\pi }{2}r^{2}h^{2}\left(t\right)\frac{dh(t)}{dt}=\mu f_{r}-\rho \frac{\pi }{4}rh^{2}\left(t\right)v$$

In Equation (6), the obtained model does not explicitly show how the model uses the laser power. Also, several process parameters and material properties should be taken into account in the model equation to understand the physics of the deposition process. Therefore, Equation (6) needs to be extended to an equation that relates the melt-pool dimension to the process parameters and material properties. Eagar and Tsai [29] developed a mathematical equation expressing a Gaussian heat distribution of the melt-pool where the pool shape was represented as a function of the operating parameter, and it is given by,

(7) $$n=\frac{Qv}{4\pi a^{2}\rho C\left[T_{m}-T\right]}$$

where *Q* is the laser power, *a* is the thermal diffusivity, *C* is the specific heat, $T_{m}$ is the melting temperature and *T* is the initial temperature of each layer. To extend Equation (7) to the bead geometry profile, Wang et al. [17] proposed a parametrization of the melt pool cross-sectional area at a steady state to the operating process parameters $\bar{n}$, such as,

(8) $$\bar{A}=\Gamma \left(\bar{n}\right)$$

where Γ is a general linear function. Wang expressed the melt pool cross-sectional area at a steady state as,

(9) $$\bar{A}=\frac{Av^{2}}{4a^{2}}$$

Following this idea, this work adopts the same assumption for the cross-sectional area in the LWAM and assumes the existence of a linear coefficient called Γ. From this assumption, Wang’s parametrization, shown in Equation (8), becomes,

(10) $$\frac{Av^{2}}{4a^{2}}=\Gamma \frac{Qv}{4\pi a^{2}\rho C\left[T_{m}-T\right]}$$

From Equation (10), the cross-sectional area *A* is derived as,

(11) $$A=\Gamma \frac{Q}{\pi \rho vC\left[T_{m}-T\right]}$$

From Equation (11), it is evident that the melt-pool area varies with the increase or decrease of the laser power, and it also varies with the variation of the velocity. Considering the melt pool’s balance Equation (1) at steady-state conditions, d/dt(ρV) = μ$f_{r}$−ρ*Av* = 0, gives μ$f_{r}$ = ρ*Av*. This means that the material transfer rate is equal to the mass rate of solidification at a steady state. Then the material transfer rate, μ$f_{r}$ can be approximated to the mass rate of solidification. Therefore, the following equation can be drawn,

(12) $$\mu f_{r}≅\rho Av$$

and thus the material transfer rate from Equation (12) is equaled to Equation (11), such as,

(13) $$\mu f_{r}≅\Gamma \frac{Q}{\pi C\left[T_{m}-T\right]}$$

Therefore, the mass balance equation becomes,

(14) $$\rho \frac{\pi }{2}r^{2}h^{2}\left(t\right)\frac{dh(t)}{dt}=\Gamma \frac{Q}{\pi C\left[T_{m}-T\right]}-\rho \frac{\pi }{4}rh^{2}\left(t\right)v$$

The obtained Equation (14) can be further extended. The extension can be possible with Pinkerton et al.’s works [30]. Pinkerton stated that the heat flowing into the melt pool is determined by considering the interaction between laser parameters and the melt pool. Pinkerton et al. expression has used the optics parameters and the power variation with respect to the z-axis to describe the system. It assumes the laser beam has an even power distribution for calculating the intensity of radiation that reaches the melt pool. The equation states that the diameter of the laser beam in a horizontal plane through the deposition point, *d*, is given in terms of the melt pool standoff, $z_{\mathrm{pool}}$, the beam diameter *D*, and the focal length of the objective lens *f*, and it is given by,

(15) $$d=D\left|\frac{f-z_{\mathrm{pool}\;}}{f}\right|$$

Further, Pinkerton investigated the relationship between the intensity and other variables. Pinkerton developed another equation that relates the intensity of the laser source *I* to other process parameters and material properties around the beam area at the melt pool, such as,

(16) $$I=\frac{4f^{2}\left(1-r_{p}\right)Q}{\pi D^{2}{\left(f^{-z}\mathrm{pool}\right)}^{2}}$$

where *Q* is the laser power and $r_{p}$ is the proportion of laser power reflected from the wire. Also, Pinkerton assumed that the heat flowing into the melt pool is proportional to the intensity of the laser source *I*, the melt pool width *w* and the melt pool’s surface absorptivity α. Therefore, the rate of heat flowing into the melt pool is expressed as,

(17) $$\dot{Q}=\frac{\pi \alpha Iw^{2}}{4}$$

Based on Pinkerton’s equations, it is now assumed that in the LWAM process, the heat flowing into the melt pool can be expressed as,

(18) $$\dot{Q}=\frac{\alpha f^{2}w^{2}\left(1-r_{p}\right)Q}{D^{2}{\left(f-z_{\mathrm{pool}\;}\right)}^{2}}$$

where α is the absorptivity of the melt pool, *f* is the focal length, *w* the width of the deposited bead, $r_{p}$ is the reflected laser power from the wire, *D* is the beam diameter of the laser source and $z_{\mathrm{pool}}$ is the standoff distance.

**Figure 3:**
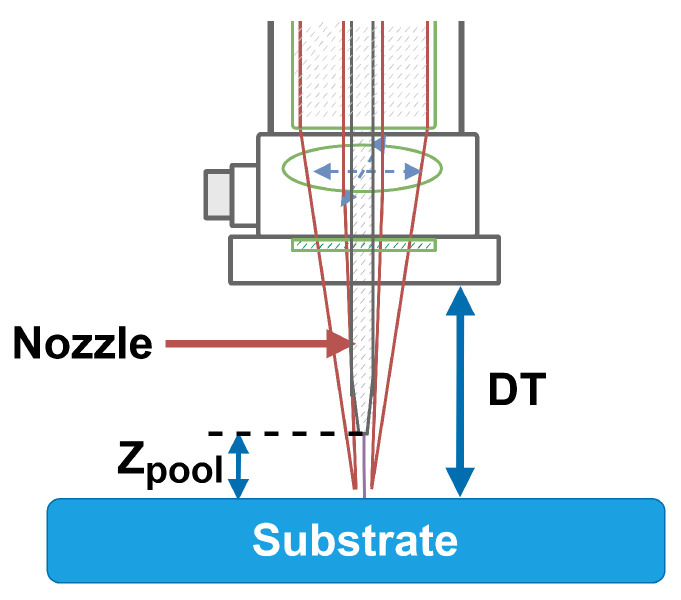
Standoff distance set-up.

In LWAM, the standoff distance ($z_{\mathrm{pool}}$) is not stated forward and well defined as in other processes, this variable will be set up, in this work, in the following manner. The $z_{\mathrm{pool}\;}$ is defined with respect to DT, which is called the working distance. In this work, we assume that $DT=z_{\mathrm{pool}}+n_{z}$, as shown in Figure 3, where $n_{z}$ is the nozzle variable that describes by the movement of the nozzle on the Z-axis.

**Figure 4:**
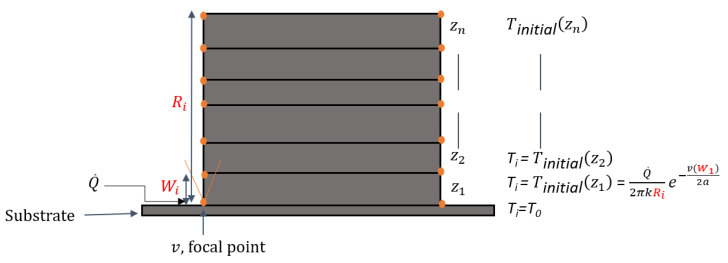
Rosenthal’s solution applied to the LWAM process.

In order to extend the Equation (19) to include more process parameters and material properties, the Rosenthal equation is used in the LWAM process [31]. It is assumed that the heat flowing into the melt pool moves from layer to layer during the deposition process. The application of Rosenthal’s equation at each layer edge is assumed to be,

(19) $$T\left(Z_{i}\right)=T_{i-1}+\frac{\dot{Q}}{2\pi kR_{i}}e^{-\frac{v\left(W_{i}\right)}{2a}}$$

where $T\left(Z_{i}\right)$ is the initial temperature at layer $Z_{i}$, $T_{i-1}$ is the temperature of the preceding layer with i=1 to *n*; *n* is the last layer, *k* is the thermal conductivity, $R_{i}$ is the length of the part, $W_{i}$ is the theoretical layer thickness, *v* is the scan speed and *a* is the thermal diffusivity, as shown in Figure 4.

Substituting Equation (18) into Equation (19) will change the temperature distribution at each layer edge (the orange points in Figure 4) while including more process variables ($z_{pool}$, *f*, *D*, *v*) and more material properties ( α, *k*, *a* ) to the LWAM process for a fair physical description of the process, as described by,

(20) $$T\left(z_{i}\right)=T_{i-1}+\frac{\alpha f^{2}w^{2}\left(1-r_{p}\right)Q}{2\pi kR_{i}\left(D^{2}{\left(f-z_{pool}\right)}^{2}\right)}e^{-\frac{v\left(W_{i}\right)}{2a}}$$

Finally, the mass balance equation from Doumanidis (1) becomes an extended dynamics multivariable equation applied to the LWAM process, given by,

(21) $$\rho \frac{\pi }{2}r^{2}h^{2}\left(t\right)\frac{dh(t)}{dt}+\rho \frac{\pi }{4}rh^{2}\left(t\right)v=\Gamma \frac{Q}{\pi C\left(T_{m}-T_{i-1}-\frac{\alpha Qf^{2}w^{2}\left(1-r_{p}\right)}{2\pi kR_{i}\left(D^{2}{\left(f-z_{pool}\right)}^{2}\right)}e^{-\frac{v\left(W_{i}\right)}{2a}}\right)}$$

Equation (21) calculates the melt pool height for the LWAM process while taking into consideration several process variables and material properties involved in the complex process. This equation includes several variables that can be used for simulation purposes to understand their influence on bead geometry. Further, this equation can be used in control system design to regulate one or more deposition process parameters, as will be discussed in Section 3.

## 3. Model Predictive Controller Design

In this section, a controller is designed based on the developed numerical model derived in Section 2, Equation (21). Since the created model has many variables with constraints, and some of the variables can be used simultaneously as inputs, a Model Predictive Controller (MPC) is proposed to obtain a good process deposition efficiency by controlling the layer height.

MPC is a model-based control strategy. The MPC calculates the optimal input value by predicting the future state and the outputs over a prediction horizon with constraints based on the process model and the current state value. Therefore, this section aims to use the MPC controller to predict and compensate the layer height eventual error during the LWAM process. The physics-based model developed in Section 2 provided 5 inputs that can be used singularly or combined simultaneously. To design the controller, the developed model is first linearized using Taylor’s series expansion and the linear state-space model is used as the process plant of the MPC controller.

### 3.1. Model Linearization

The linearization process has been applied in many domains where the non-linear model is difficult to solve [32,33,34,35,36]. In Section 2, the developed model is first linearized using Taylor’s series expansion and the Jacobian linearization process. In the linearization process, the gradient of the nonlinear equation for all process parameters and material properties variables is evaluated in order to create a linear representation at the evaluation point.

In the developed model (given by Equation (21)), the main process parameters are the temperature, the laser power, the travel speed, the wire-feed rate and the standoff distance. Therefore, these parameters are selected as inputs. The state vector is h(t) and the inputs are $T_{i-1}\left(t\right)$, Q(t), v(t), $W_{i}$, and $z_{pool}$, and it is represented in the following equation,

(22) $$\frac{dh(t)}{dt}=\dot{h}=\frac{2\Gamma Q}{\pi^{2}Ch^{2}r^{2}\rho \left(-\frac{Qf^{2}w^{2}\alpha \left(1-r_{p}\right)e^{-\frac{W_{i}v}{2a}}}{2\pi R_{i}D^{2}k{\left(f-z_{\mathrm{pool}\;}\right)}^{2}}+T_{m}-T_{i-1}\right)}-\frac{v}{2r}$$

This equation is linearized by using a Taylor series expansion as the following,

(23) $$\frac{dh}{dt}=f\left(h,u\right)\approx f\left(\bar{h},\bar{u}\right)+{\left.\frac{\partial f}{\partial h}\right|}_{\bar{h},\bar{u}}\left(h-\bar{h}\right)+{\left.\frac{\partial f}{\partial u}\right|}_{\bar{h},\bar{u}}\left(u-\bar{u}\right)$$

where, $\bar{h}$ and $\bar{u}$ are the steady-state values of the state and the inputs; hence, $f(\bar{h},\bar{u})=0$.

The linearized expression is further simplified using deviation variables; defined as $h^{\prime }=h-\bar{h}$ and $u^{\prime }=u-\bar{u}$. The deviation variables are the change in the steady-state conditions, and the derivatives of the deviation variable is given as, $\frac{dh^{\prime }}{dt}=\frac{dh}{dt}-\frac{d\bar{h}}{dt}$ and $\frac{du^{\prime }}{dt}=\frac{du}{dt}-\frac{d\bar{u}}{dt}$. The partial derivatives at steady-state are zero $\frac{d\bar{h}}{dt}=0$, $\frac{d\bar{u}}{dt}=0$. Therefore $\frac{dh^{\prime }}{dt}=Ah^{\prime }+Bu^{\prime }$, where *A* and *B* are the partial derivatives of f(h,u) at steady-state conditions. Now, based on the fact that, the general state-space form of a Linear Time Invariant (LTI) system is given as,

(24) $$\begin{array}{cc} \; & \dot{x}=Ax+Bu\\ \; & y=Cx+Du \end{array}$$

where $x\in R^{n}$ is the state, $\dot{x}\in R^{n}$ is the state derivative, $y\in R^{p}$ is the output, $u\in R^{m}$ is the input. *A* is the state matrix, *B* is the input matrix and *C* the output matrix. *D* is the matrix showing the influence of the inputs on the output. The dimensions of the matrix are $A\in R^{n\times n}$$B\in R^{n\times m}$$C\in R^{p\times n}$$D\in R^{p\times m}$ with *m* the inputs, *n* the states and *p* the outputs.

The linearized equation in linear state space matrix formed with five inputs, one state, and one output, is given by (see Appendix A for more details),

(25) $$A=\left[{\left.\frac{\partial f}{\partial x}\right|}_{\bar{x},\bar{u}}\right]$$

(26) $$B=\left[{\left.\frac{\partial f}{\partial T_{i-1}}\right|}_{\bar{h},\bar{u}}+{\left.\frac{\partial f}{\partial \bar{Q}}\right|}_{\bar{h},\bar{u}}+{\left.\frac{\partial f}{\partial v}\right|}_{\bar{h},\bar{u}}+{\left.\frac{\partial f}{\partial W_{i}}\right|}_{\bar{h},\bar{u}}+{\left.\frac{\partial f}{\partial z_{pool}}\right|}_{\bar{h},\bar{u}}\right]$$

(27) C=1andD=0

### 3.2. Controller Design

**Figure 5:**
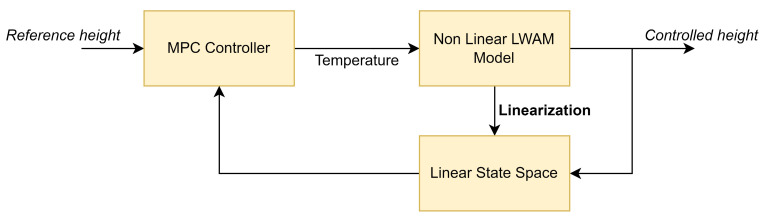
MPC schematic diagram for the layer height control in the LWAM process.

The control layer of the MPC problem is formulated as a quadratic optimization problem that is solved at each time *k* to determine the control actions considering that the values of state and inputs are known. The following cost function is used to represent the control problem:

(28) $$\begin{array}{cc} \min_{u} & \;J\left(x\left(k\right),u\left(.\right)\right)=\sum_{i=1}^{H_{p}}{\left\{\frac{w_{i}^{y}}{s^{y}}\left[r\left(k+i|k\right)-y\left(k+i|k\right)\right]\right\}}^{2}\\ \;s.T.\; & \\ & u_{lb}\leq u\left(k+j|k\right)\leq u_{ub}\\ & y_{lb}\leq y\left(k+i|k\right)\leq y_{ub}\\ & \forall \;i\in \left\{1,\ldots ,H_{p}\right\}\;and\;j\in \left\{0,\ldots ,C_{h}\right\}. \end{array}$$

where, *k* is the current control interval, $H_{p}$ is the prediction horizon, $C_{h}$ is the control horizon, where $\epsilon_{k}$ is a non-negative slack variable, which quantifies the worst-case constraint violation. y(k+i|k), r(k+i|k) are the predicted value of the plant output and the reference value at *i*th prediction horizon step, respectively. $s^{y}$ is a scale factor of the plant output, $w_{i}^{y}$ is the tuning weight for the plant output at *i*th prediction horizon. $u_{lb}$,$u_{ub}$ and $y_{lb}$, $y_{ub}$ are the low bound and upper bound of the input and output respectively. Figure 5, shows the schematic diagram of the proposed MPC controller design for the LWAM system.

**Remark** **1.** *For simplicity, in this work, only the temperature input was used as the manipulated variable for the MPC controller design. More specifically, the height controller applied a generalized MPC algorithm that takes the feedback signals into the control action to improve the system performance by controlling the temperature input. This control system configuration is able to stabilize layer growth by avoiding over-building and compensating for under-building through control of the process heat input in order to have a fixed layer height [37].*

For this study, the sample time $T_{s}$ has been chosen to be 10% of the rise time. The prediction horizon $P_{h}$ has been defined to include 20 sample times during the transient system response. The control horizon $C_{h}$ was considered to stay around 10–20% of the prediction horizon. The constraints are set to around 1/10th of the nominal constraint values (for more details see e.g., [38]).

## 4. Simulation and Experimental Results

**Figure 6:**
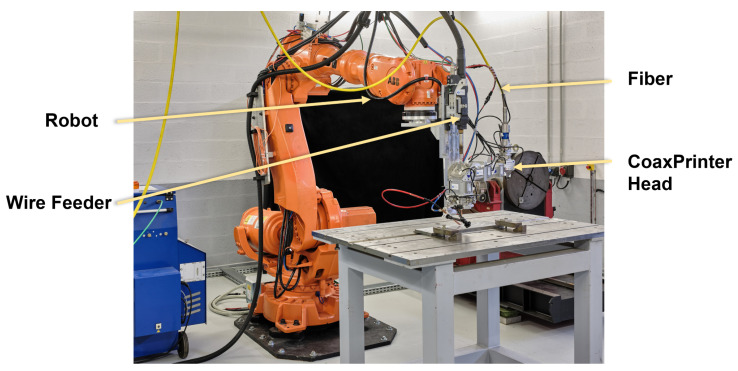
Experimental Setup of LWAM platform at Institut de Soudure.

**Figure 7:**
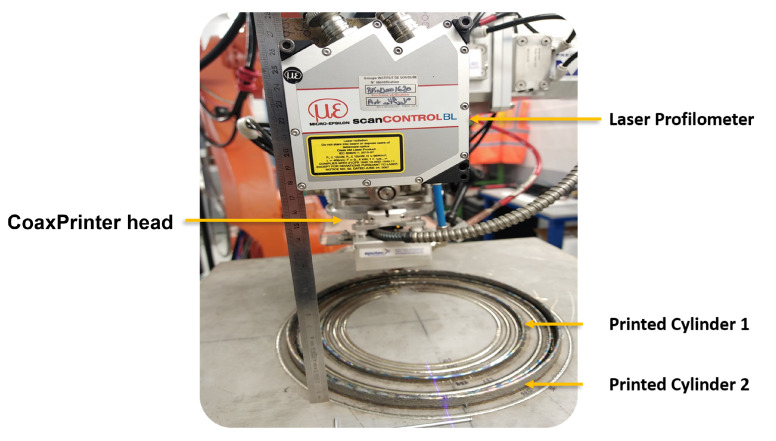
Profilometer set-up for data acquisition.

Experimental tests for this study were carried out on the laser platform at Institut de Soudure. Tests were run by a robotized laser wire-feed system. An ABB 7-axis poly-articulated robot was used to provide the kinematics of the process. A fibre laser source, IPG Photonics, of 10 kW was used as the heat source. The system also used a CoaxPrinter laser processing head to deliver the laser beam and the filler wire to the processing zone. Figure 6 shows the actual LWAM system setup.

### 4.1. Numerical Model Validation

For validating the physics-based model, derived in Section 2 and given by Equation (21), two metallic hollow cylinders were deposited. It is worth mentioning that the cylinder shape was chosen as a deposition object, based on the fact that it is more convenient for a continuous and clear height profile acquisition due to the configuration of the laser head and the placement of the profilometer, as shown in Figure 7.

The deposition process parameters for both cylinders are summarized in Table 1. Furthermore, the process variables and material properties of Inconel 718 with their definitions are shown in Table 2 and Table 3, respectively.

**Figure 8:**
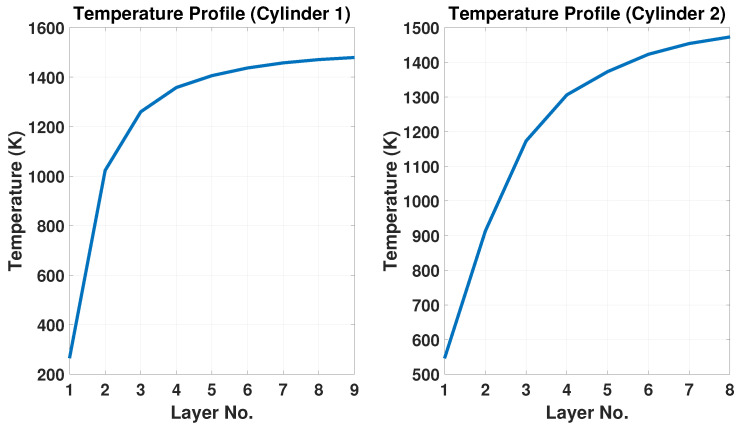
Temperature profile of cylinder 1 and cylinder 2.

Cylinder 1 has a diameter of 180 mm and cylinder 2 has a diameter of 250 mm. The multi-step input trajectory of the temperature is 9 intervals for the first cylinder and 8 intervals for the second cylinder. The mean temperature is averaged for each layer and it has been considered as an input profile for the model validation, as shown in Figure 8.

The observation in Figure 8 can be explained by the high cooling and thermal dissipation of the first layers on the substrate. The layer temperature increases significantly in the early layers and increases slowly at the final layers, where it starts to reach a steady-state point. Therefore, as the layers increase, the thermal dissipation reduces and tends to reach the steady-state (layer 9 for cylinder 1 and 8 for cylinder 2). It is observed that when the temperature drops significantly at the earlier layers and less at the last layers, desired layer heights can be obtained. This observation matches our assumption in Remark 1 and shows that the accumulation of height errors could be decreased by properly controlling the temperature input while printing the final product.

For each layer, bead geometry information of the deposited layer is collected using a scanCONTROL 2950-100/BL laser profilometer. The measurement range of the device is up to 265 mm on the z-axis, and the measurement rate is up to 2,560,000 points/s. The measuring range on the x-axis is up to 143.5 mm and the accuracy of the measurement is 12 µm.

**Figure 9:**
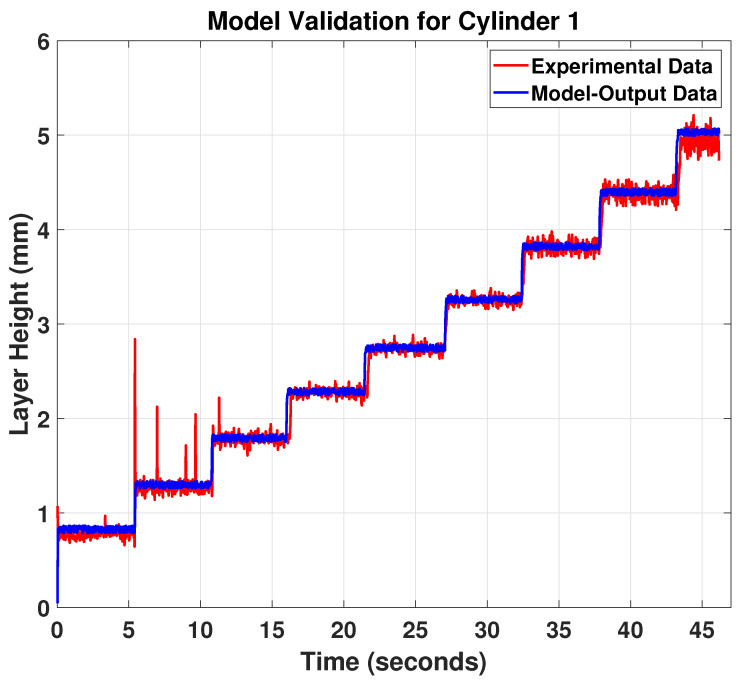
Model validation for cylinder 1.

**Figure 10:**
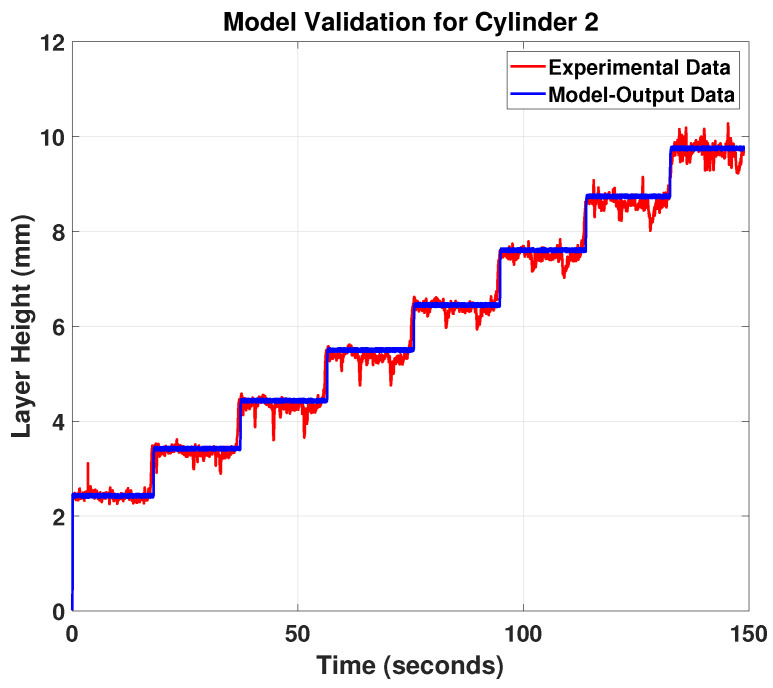
Model validation for cylinder 2.

For this study, the profilometer laser is projected near the melt pool and close to the hot deposited layer to measure the bead geometry (see Figure 7). The target material, which is the hot Inconel 718, is a red-hot glowing metal. It has a wavelength of 1000 nm and emits a high proportion of light at the wavelengths in which the laser operates. Blue laser light was used at a shorter wavelength of 405 nm, far from the visible spectrum red part. This means that the blue laser light is unaffected by the emitted light and can provide stable signals. The data acquisitions of the two cylinders with model validation outputs are shown in Figure 9 and Figure 10.

The results in Figure 9 and Figure 10 show that the proposed model predictions (blue curve) for the melt-pool height follow the experimental measurements (red curve) very well, and both the predictions and measurements show a clear pattern of step changes, correlating to the step changes of the printed layer height. Based on this obtained model, an MPC feedback controller can be designed to track the melt-pool height trajectory using the temperature input to improve the final printed product. However, some fluctuations can be seen in the experimental data. This can be explained by the vibrations caused by the robot’s movement while collecting the data using the laser profilometer. Furthermore, the surface roughness of the printed cylinder provides additional noise to the bead height measurements. Nonetheless, this noise and fluctuation of the collected data are common in the Metal Additive Manufacturing processes (see e.g., [17]) and it doesn’t affect the influence of the general dynamic behaviour of the system.

### 4.2. Layer Height Controller

In this section, the proposed MPC is tested using the process parameters and material properties of Inconel^®^ 718 [43] that are given in Table 4. The linearized model discussed in Section 3.1, for these parameter values, is given by the following state-space matrices, A=[−0.2262]; *A* is the system matrix. *B* = [8.77 × 10^−8^, 0.0001338, 1.815 × 10^−7^, −0.01047, −0.1466]; *B* is the input matrix where rows 1 to 5 represent the power input, the standoff input, the temperature input, the wire-feed rate and the travel speed, respectively. However, for this work, and as mentioned in Section 3, only the temperature is selected and simulated as the input of the MPC controller. Finally, the output matrix C=1000 and the feed-forward matrix D=0.

The simulation is performed in the MPC Designer toolbox in the Matlab^®^ software. The input temperature is constrained to 273 Kelvin as the minimum and 1450 Kelvin as the maximum. It is worth mentioning that; the temperature of the deposited bead was measured layer by layer using a thermal camera during the experimental deposition. It was observed that the temperature signals of the deposited beads displayed a measured temperature of 1400 to 1450 Kelvin, and hence, the maximum input temperature in this work was chosen to be 1450 Kelvin. The height output constraints are 0.75 mm for the minimum and 0.9 mm for the maximum. The increments rate is 100 Kelvin. The chosen sampling time is $T_{s}$ = 0.1 s, corresponding to around every 3 bead profiles. The prediction horizon is $H_{p}=15$ and the selected control horizon is $C_{p}=3$. The tuning weight matrices of the input are selected to be 0.2 in order to balance the controller response and the temperature input movement. The input weight matrix $Q_{i}$ is not specified to avoid steady-state error in the output. The output weight matrix is $Q_{o}=5$, so the height is kept near the reference height input. The MPC parameters are summarized in Table 5.

**Figure 11:**
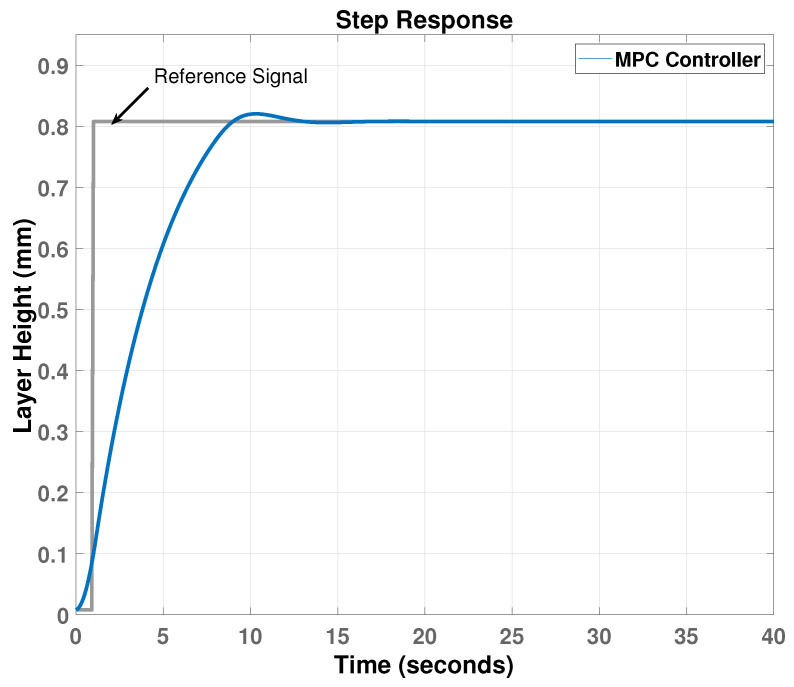
Step response of the closed-loop feedback system.

**Figure 12:**
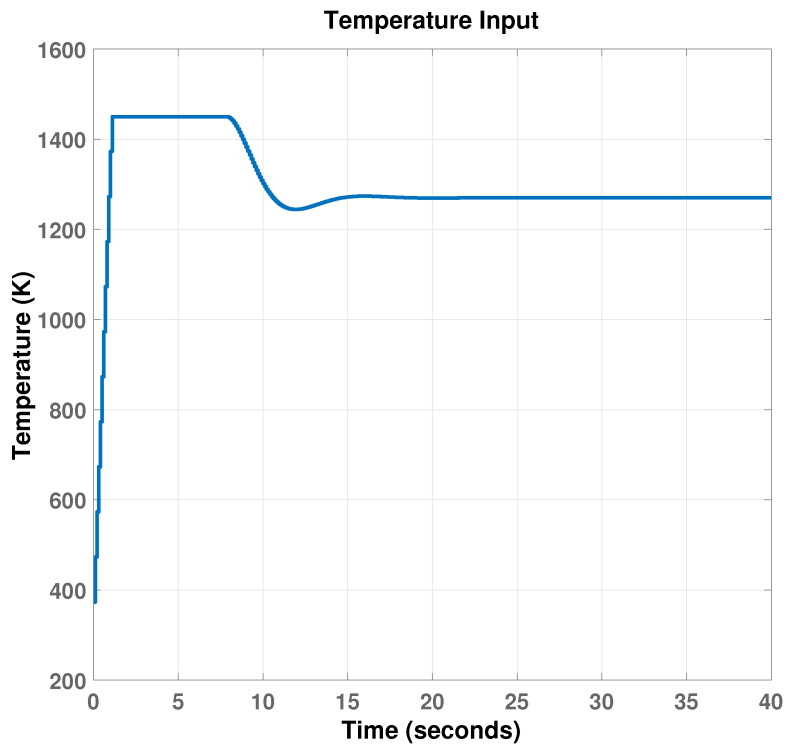
Controlled input temperature.

The simulation result in Figure 11 shows that the MPC controller can track the layer height reference with little overshoot and acceptable responding time. The input variable, which is the temperature, increases at the beginning to reach a maximum of 1450 Kelvin and then decreases to its steady-state value of 1270 kelvin, as shown in Figure 12.

## 5. Conclusions

This work developed a physics-based model of the bead geometry for the Laser Wire Additive Manufacturing process (LWAM). The developed model aimed to include known process parameters and material properties to describe the LWAM process properly. Experimental validation results were performed to validate the accuracy of the proposed model, and an MPC controller was designed. The following conclusions are drawn,

- The proposed model describes and simulates the behavior of the bead geometry in the laser wire additive manufacturing process.
- The temperature is an important input parameter and significantly influences the layer by layer deposition.
- The MPC controller can track the reference height and regulate the temperature input while keeping the parameters in their region of operation.
- The system response shows an acceptable transient response with less overshoot.

For future works, a Multi-Input Multi-Output MPC controller could be designed and implemented to control the complete deposition process. Also, a gain-scheduling can be considered to reflect the change of some material properties of Inconel 718 that changes with the temperature in the LWAM process.

## Figures and Tables

**Table 1:** Process parameters for the two deposited cylinders.

	Power (W)	Travel Speed (m/min)	Wire Feed Rate (m/min)	Standoff Distance (mm)	Number of Layers	Layer Thickness (mm)
Cylinder 1	2700	2.0	2.4	1.5	9	0.7
Cylinder 2	2100	0.6	2.1	1.5	8	1.2

**Table 2:** Process variables and material properties of Inconel 718.

Symbols	Parameters Name	Parameters Value	Sources	Units
** Γ **	Gain	Variable	Experiments	Unitless
***Q***	Power	Variable	Experiments	W
***C***	Melt specific heat	760–800	[39]	J/(kg K)
** $T_{m}$ **	Melting temperature	1570	[39]	K
** $T_{i-1}$ **	Temperature of the preceeding layer	Variable	Experiments	K
** α **	Absorptivity of the melt pool	0.5	[40]	Unitless
***f***	Focal length of the objective lens	160 × 10^−3^	laser beam is focess	m
***w***	Melt pool width	Variable	Experiments	m
** $r_{p}$ **	Proportion of laser power	0.7	[41]	Unitless
***k***	Thermal conductivity	33	[39]	W/m·K
** $R_{i}$ **	Height of the product	Variable	Experiments	m
***D***	Laser beam diameter of the laser	3.95 × 10^−3^	Relative to the printed head	m
** $z_{pool}$ **	Standoff distance	Variable	Experiments	*m*
***v***	Travel speed of the robot	Variable	Experiments	m/s
** $W_{i}$ **	Layer thickness	Variable	Experiments	m
***a***	Thermal diffusivity	a=k/(C/ρ)	[42]	m^2^/s
** ρ **	Melt pool density	Variable	material	kg/m^3^
***r***	Width over height ratio	Variable	Experiments	Unitless
***h***	Melt pool height	Output	Calculated using the model	m

**Table 3:** Summary of the definition of the parameters.

Parameter	Definition
** Γ **	Constant gain
***Q***	Input laser power
***C***	Quantity of heat needed to increase the temperature 1 K per unit mass (kg)
** $T_{m}$ **	Melting temperature of the material
** $T_{i-1}$ **	Temperature of the layer where a new deposition will be done
** α **	The degree to which the material absorbs the laser power
***f***	The distance from the last lens to the point at which the laser beam is focussed
***w***	The measured width of a deposited bead
** $r_{p}$ **	Reflected laser power from the material wire
***k***	The rate at which the heat is transferred by conduction through a unit cross-section area of material
** $R_{i}$ **	Total height of the part to be produced
***D***	The diameter of the focuses laser beam
** $z_{pool}$ **	Distance from the substrate to the nozzle tip
***v***	Deposition speed
** $W_{i}$ **	Theoretical layer thickness
***a***	The ability of the material to conduct thermal energy thermal energy
** ρ **	Density of the material - the mass of a unit volume of the material
***r***	The ratio of the width to the height
***h***	Height of the deposited beads

**Table 4:** Parameters values used for MPC controller testing.

Parameter	Value	Parameter	Value	Parameter	Value
** Γ **	0.15	***D***	1.5 × 10^−3^ (m)	** $W_{i}$ **	0.7 (m)
***C***	800 (J/(kg K)	** $T_{m}$ **	1570 (K)	** $T_{0}$ **	273 (K)
***f***	160 × 10^−3^ (m)	***w***	2.8 × 10^−3^ (m)	** $r_{p}$ **	0.7
***r***	2.33 × 10^−3^ (m)	***k***	33 (W/m·K)	** ρ **	8145 (kg/m^3^)
** $R_{i}$ **	12 × 10^−3^ (m)	***a***	5.0 × 10^−6^ (m^2^/s)	** α **	0.5 (m)

**Table 5:** MPC parameters for layer height control.

MPC Parameter	Min Value	Value	Max Value
Sampling time ($T_{s}$ in s)	-	0.1	-
Prediction horizon ($P_{h}$)	-	15	-
Control horizon ($C_{h}$)	-	3	-
Input constraint (K)	273	-	1450
Output constraint (mm)	0.75	-	0.9
Input weight	-	0	-
Output weight (mm)	-	5	-

## Data Availability

Not applicable.

## Abbreviations

The following abbreviations are used in this manuscript:

MDPI	Multidisciplinary Digital Publishing Institute
DOAJ	Directory of open access journals
TLA	Three letter acronym
LD	Linear dichroism

## Appendix A

Based on the linearized state-space equations and more specifically Equations (25) and (26), the computed matrices *A* and *B*, for the LWAM process are given as follows,

(A1) $$\begin{array}{cc} A & =-\frac{4\Gamma Q}{\pi^{2}Ch^{3}r^{2}\rho \left(-T_{i}+T_{m}-|\frac{Qf^{2}w^{2}\alpha \left(1-r_{p}\right)e^{-\frac{W_{ij}}{2a}}}{2\pi R_{i}D^{2}{\left(f-z_{pool}\right)}^{2}}\right)} \end{array}$$

(A2) $$\begin{array}{cc} B_{11} & =\frac{2\Gamma Q}{{\left.\pi^{2}Ch^{2}r^{2}\rho {\left(-T_{i}+T_{m}-\frac{Qf^{2}w^{2}a\left(1-r_{p}\right)e}{2\pi R_{i}D^{2}{\left(f-z_{pool}\right)}^{2}}\right)}^{-\frac{W_{ij}v}{2a}}\right)}^{2}} \end{array}$$

(A3) $$\begin{array}{cc} B_{12} & =\frac{2\Gamma }{\pi^{2}Ch^{2}r^{2}\rho \left(-T_{i}+T_{m}-\frac{Qf^{2}w^{2}\alpha \left(1-r_{p}\right)e}{2\pi R_{i}D^{2}k{\left(f-z_{pool}\right)}^{2}}\right)}\\ \; & +\frac{\Gamma Qf^{2}w^{2}\alpha \left(1-r_{p}\right)e^{-\frac{W_{ij}v}{2a}}}{{\left.\pi^{3}CR_{i}h^{2}r^{2}\rho D^{2}k{\left(f-z_{pool}\right)}^{2}{\left(-T_{i}+T_{m}-\frac{Qf^{2}w^{2}\alpha \left(1-r_{p}\right)e}{2\pi R_{i}D^{2}k{\left(f-z_{pool}\right)}^{2}}\right)}^{-\frac{W_{ij}v}{2a}}\right)}^{2}} \end{array}$$

(A4) $$\begin{array}{cc} B_{13} & =-\frac{1}{2r}-\frac{W_{ij}\Gamma Q^{2}f^{2}w^{2}\alpha \left(1-r_{p}\right)e^{-\frac{W_{ij}v}{2a}}}{{\left.2\pi^{3}CR_{i}h^{2}r^{2}\rho D^{2}ak{\left(f-z_{pool}\right)}^{2}{\left(-T_{i}+T_{m}-\frac{Qf^{2}w^{2}a\left(1-r_{p}\right)e}{2\pi R_{i}D^{2}k{\left(f-z_{pool}\right)}^{2}}\right)}^{-\frac{W_{ij}v}{2a}}\right)}^{2}} \end{array}$$

(A5) $$\begin{array}{cc} B_{14} & =-\frac{g\Gamma Q^{2}vf^{2}w^{2}\alpha \left(1-r_{p}\right)e^{-\frac{W_{ij}v}{2a}}}{{\left.2\pi^{3}CR_{i}h^{2}r^{2}\rho D^{2}ak{\left(f-z_{pool}\right)}^{2}{\left(-T_{i}+T_{m}-\frac{Qf^{2}w^{2}\alpha \left(1-r_{p}\right)e}{2\pi R_{i}D^{2}k{\left(f-z_{pool}\right)}^{2}}\right)}^{-\frac{W_{ij}v}{2a}}\right)}^{2}} \end{array}$$

(A6) $$\begin{array}{cc} B_{15} & =\frac{2\Gamma Q^{2}f^{2}w^{2}\alpha \left(1-r_{p}\right)e^{-\frac{W_{ij}v}{2a}}}{\pi^{3}CR_{i}h^{2}r^{2}\rho D^{2}k{\left(f-z_{\mathrm{pool}\;}\right)}^{3}{\left(-T_{i}+T_{m}-\frac{Qf^{2}w^{2}\alpha \left(1-r_{p}\right)e^{-\frac{W_{ij}v}{2a}}}{2\pi R_{i}D^{2}k{\left(f-z_{\mathrm{pool}\;}\right)}^{2}}\right)}^{2}} \end{array}$$
